# The dosing of aerobic exercise therapy on experimentally-induced pain in healthy female participants

**DOI:** 10.1038/s41598-019-51247-0

**Published:** 2019-10-16

**Authors:** Anna M. Polaski, Amy L. Phelps, Kimberly A. Szucs, Austin M. Ramsey, Matthew C. Kostek, Benedict J. Kolber

**Affiliations:** 1 Department of Biological Sciences, Duquesne University, Pittsburgh, Pennsylvania United States; 2 Chronic Pain Research Consortium, Duquesne University, Pittsburgh, Pennsylvania United States; 3 Palumbo Donahue School of Business, Statistics, Duquesne University, Pittsburgh, Pennsylvania United States; 4 Department of Occupational Therapy, Duquesne University, Pittsburgh, Pennsylvania United States; 5 Department of Physical Therapy, Duquesne University, Pittsburgh, Pennsylvania United States

**Keywords:** Chronic pain, Somatic system

## Abstract

Knowledge of efficacious dosing respective to exercise type and pain condition is extremely limited in the literature. This study aimed to determine the impact of dose of moderate intensity treadmill walking on experimentally-induced pain in healthy human participants. Forty females were divided into 4 groups: control (no exercise), low dose exercise (3×/wk), moderate dose exercise (5×/wk) or high dose exercise (10×/wk). Over a 7-day period, subjects performed treadmill walking during assigned exercise days. Both qualitative and quantitative measures of pain were measured at baseline, during the trial, and 24 hrs post-final intervention session via sensitivity thresholds to painful thermal and painful pressure stimulation. Significant effects of treatment were found post-intervention for constant pressure pain intensity (p = 0.0016) and pain unpleasantness ratings (p = 0.0014). Post-hoc tests revealed significant differences between control and moderate and control and high dose groups for constant pressure pain intensity (p = 0.0015), (p = 0.0094), respectively and constant pressure pain unpleasantness (p = 0.0040), (p = 0.0040), respectively. Moderate and high dose groups had the greatest reductions in ratings of pain, suggesting that our lowest dose of exercise was not sufficient to reduce pain and that the moderate dose of exercise may be a sufficient starting dose for exercise-based adjuvant pain therapy.

## Introduction

Exercise, or planned physical activity, has effects on nociception and pain in humans^1,2^. Exercise has been increasingly reported as a preferred intervention for the management of most chronic pain syndromes^2^, which has been estimated to affect 116 million American adults^3^. Exercise as a viable therapy is particularly valuable given that the existing pharmacological options for chronic pain include severe limitations due to side-effects, abuse potential, and overall efficacy^4,5^. Physical activity has also been linked with reduced symptoms of depression and anxiety, indicating that exercise could be especially beneficial in the circumstance where chronic pain is comorbid with psychiatric illness^6,7^. Overall, while evidence purports exercise to be at least relatively therapeutic in chronic pain^8^, adherence to exercise programs reveals a number of issues. These include limited access to equipment and training for safe exercise and the lack of dosing information specific to exercise type and chronic pain population. The issue of dose is particularly noteworthy due to the clear emphasis and importance of dosing for pharmacological development.

Interestingly, there is a notable lack of trials in the exercise and pain literature examining different doses of exercise training in a distinct patient population or in healthy control participants. In fact, the American College of Sports Medicine (ACSM) does not have a specific exercise recommendation for pain. The ACSM’s Position Stand (2011) recommends 30 minutes of moderate-intensity walking exercise, five days per week (or 150 MET minutes) to sufficiently sustain adequate cardiorespiratory, musculoskeletal, and neuromotor fitness for healthy adults^9^. Long-term maintenance of this exercise dose regimen would be ideal. This recommendation may be too high of a starting point for individuals with chronic pain, especially in the context of movement-induced pain and movement-associated fear avoidance behavior^10,11^. Therefore, it is crucial that the field critically examine the most suitable dose of exercise to reduce pain. Furthermore, there is a notable lack of pain studies performed in females, which makes this investigation especially relevant.

A limited number of studies have tested the effect of multiple doses of acute or single bouts of exercise on pain perception in healthy participants^12–17^, and only one recent meta-analysis has evaluated dose in the context of repeat exercise training on chronic pain^18^. As a starting point for evaluating this gap in knowledge, this study investigated the effect of three different doses of repeated moderate intensity walking exercise: low (3×/week), moderate (5×/week) and high (10×/week) compared to a sedentary control on sensitivity to acute noxious stimuli in healthy female participants. We hypothesized that moderate intensity exercise would reveal dose-related effects in subjects’ sensitivity to acutely applied noxious stimuli.

## Results

### Participant characteristics

Forty-one healthy female volunteers participated in this study. Recruitment began February 2015 and ended June 2017. Subject characteristics are presented in Table 1. 1 subject dropped out of the study after 3 days (data not included). One-way ANOVA revealed no significant group differences for any of the demographic variables. A chi-square test of independence was calculated comparing handedness of subjects with treatment group. A significant interaction was found (χ^2^(6) = 16.229, p = 0.013).

**Table 1:** Participant characteristics. Data are mean (SD).

	Control (n = 10)	Low dose (n = 10)	Moderate dose (n = 10)	High dose (n = 10)	All (n = 40)	ANOVA, P
Age (yrs)	22.7 (3.7)	20.7 (0.7)	22.3 (3.0)	20.6 (1.3)	21.6 (2.6)	0.154
BMI	22.1 (1.8)	21.8 (2.0)	22.8 (2.4)	21.9 (2.1)	22.2 (2.0)	0.717
Resting HR (bpm)	76.7 (13.3)	72.2 (12.6)	75.0 (23.3)	71.3 (11.5)	74.0 (15.9)	0.892
Resting BP_s_ (mmHg)	107.6 (10.6)	116.3 (9.1)	122.8 (13.7)	117.2 (11.0)	116.2 (12.4)	0.055
Resting BP_d_ (mmHg)	71.2 (7.2)	74.1 (10.5)	78.8 (11.4)	76.7 (6.5)	75.5 (9.1)	0.316
IPAQ (MET-min/wk)	5492.1 (25550.9)	3468.1 (2412.0)	3967.0 (2166.3)	3348.2 (2660.0)	4068.8 (2511.5)	0.203
SIAS	18.1 (8.1)	11.9 (8.0)	11.8 (8.9)	10.4 (6.4)	13.1 (8.2)	0.149

Abbreviations: BMI, body mass index; BP_s_, systolic blood pressure; BP_d_, diastolic blood pressure; IPAQ, International Physical Activity Questionnaire; SIAS, Social Interaction Anxiety Scale.

### Effect of exercise dose on quantitative sensory testing

#### Primary outcome: Intervention effects on QST perceived pain

**Figure 1:**
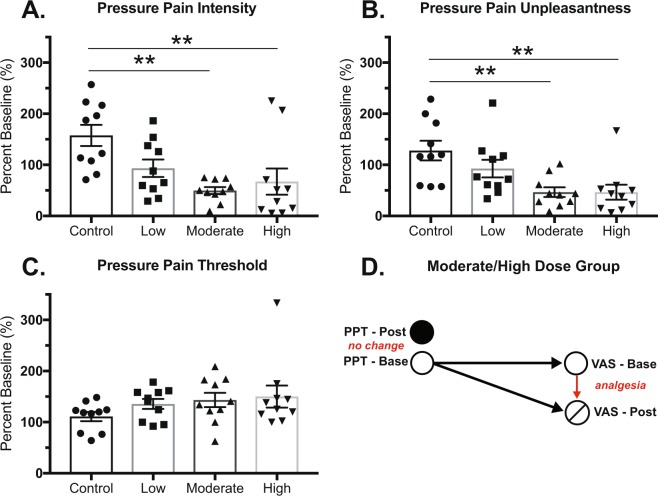
The effects of exercise intervention on qualitative and quantitative measures of pressure pain. (**A**) Percent baseline pressure pain intesnity (VAS) in the forearm was significantly different from the control group for moderate and high dose groups. (**B**) Percent baseline pressure pain unpleasantness (VAS) in the forearm was significantly different from the control group for the moderate and high dose groups. (**C**) There were no significant different between groups for percent baseline pressure pain threshold as meausred in the forearm. Tukey post-hoc significant differences between groups at **p < 0.01. Data shown as mean +/− SEM. (**D**) Model representation of average pressure pain thresholds (PPT) compared to their subsequent pain intensity rating (VAS) at baseline (Base) and post-intervention (Post) for the moderate and high groups.

We first analyzed the impact of exercise dosing on baseline versus post-intervention QST on each subject’s calf and forearm. Body site specific data for the primary outcome variables are shown in Table 2. Interestingly, the most robust dose effect of exercise was found for pressure pain on the forearm. Although there was no significant effect of treatment for pressure pain threshold for the forearm (p = 0.262), a significant effect of treatment was found for constant pressure VAS pain intensity (p = 0.0016) and constant pressure pain VAS unpleasantness (p = 0.0014), with exercise generally lowering the perceived VAS compared to the control group. Tukey post-hoc tests revealed significant differences between the control and moderate dose groups (p = 0.0015) and control and high dose groups (p = 0.0094) for constant pressure pain intensity and significant differences between control and moderate dose groups (p = 0.0040) and control and high dose groups (p = 0.0040) for constant pressure pain unpleasantness (Fig. 1A–C). Figure 1D shows a visual comparison of the apparent paradoxical impact of exercise on VAS measurements (i.e. perception), but not on pain threshold (i.e. sensation) for the forearm.

**Table 2:** Intervention effects on QST.

QST	ANOVA/Rank Sum, P	
	*Forearm*	*Calf*
Mechanical Sensitivity	0.713	0.673
Constant Heat Pain VAS *Intensity*	0.882	0.183
Constant Heat Pain VAS *Unpleasantness*	0.813	0.304
Radiant Heat Sensitivity	0.890	0.445
Radiant Heat Pain	0.803	0.867
Pressure Pain Threshold	0.262	0.883
Constant Pressure VAS *Intensity*	**0.002	0.958
Constant Pressure VAS *Unpleasantness*	**0.001	0.791

**p < 0.01.

#### Secondary outcomes: Acute effects of intervention

**Figure 2:**
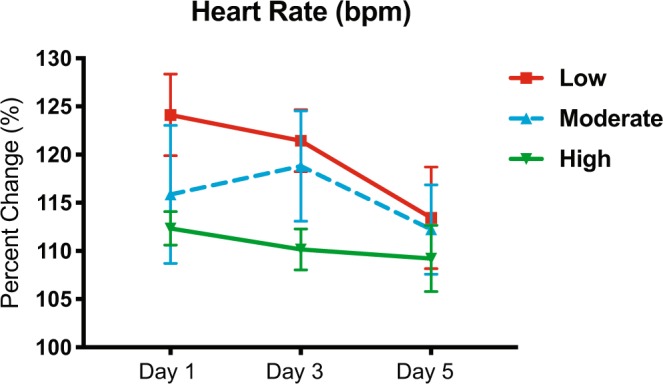
Acute effects of exercise on HR. No significant effect of treatment was found for exercise groups on percent change in HR. Data shown as mean +/− SEM.

In addition to the primary outcomes of this trial, we also evaluated a number of secondary outcomes related to physiology, fitness, and pain-effect. First, two-way ANOVA found no significant effect of treatment for the exercise groups on percent change in HR (p = 0.295; 3 subjects data unavailable due to equipment failure) (Fig. 2) nor for percent change in Borg RPE (p = 0.615; 1 subject data unavailable) over days 1, 3 and 5. Next, we evaluated the impact of the exercise dosing intervention on acute pain testing. This was done 5 min and 30 min immediately following exercise sessions on days 1, 3, and 5. For the high dose group, these measurements were taken following the second exercise session for each day. Since pain measurements at 5 min and 30 min represent two distinct dependent measures, we first analyzed these data using a MANOVA. A two-way MANOVA was calculated examining the effect of treatment (control, low, moderate, and high) and time (days 1, 3 and 5) on 5 min (dependent measure 1) or 30 min (dependent measure 2) QST outcome per body site. No significant effects were found. Overall, these data indicate that there is minimal association between the treatment or time and the relationship between the dependent variables (5 min versus 30 min post-exercise testing).

**Figure 3:**
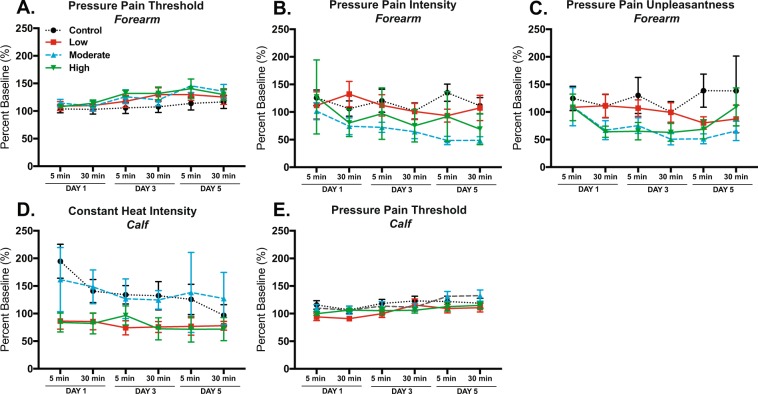
Acute effects of exercise on QST for the forearm and calf. Results of univariate tests of between-subjects effects. (**A**) Acute effect of intervention on pressure pain threshold for the forearm, (p = 0.006) for DAY at 5 min time point. (**B**) Pressure pain intensity for the forearm, (p = 0.007) for Treat at 30 min time point. (**C**) Pressure pain unpleasantness for the forearm, (p = 0.005) for Treat at 5 min time point and (p < 0.001) for Treat at 30 min time point. (**D**) Constant heat intensity for the calf, (p = 0.004) for Treat at 30 min time point. (**E**) Pressure pain threshold for the calf, (p = 0.006) for Treat at 5 min time point and (p = 0.007) for DAY at 30 min time point. Data shown as mean +/− SEM.

Next, we evaluated the impact of exercise dose separately on the 5 min and 30 min dependent measures. For the forearm, results of univariate ANOVAs on secondary outcome QST are shown in Table 3. We found statistically significant analgesic-like effects for pressure pain threshold, intensity, and unpleasantness. Five min pressure pain thresholds were significantly increased by day (p = 0.006; Fig. 3A). Thirty min constant pressure pain intensity was significantly improved by treatment (p = 0.007; Fig. 3B). Both 5 min and 30 min post-exercise constant pressure pain unpleasantness were significantly improved by treatment respectively (5 min; p = 0.005), (30 min; p < 0.001) (Fig. 3C). These results are perhaps not surprising given the overall impact of exercise on pressure pain perception described above (Fig. 1).

**Table 3:** Acute effects of intervention on forearm QST.

Source	Dependent Variable	ANOVA, P
Corrected Model	PPT–%BL 5 min	0.047
	PPT–%BL 30 min	0.356
Intercept	PPT–%BL 5 min	<0.001
	PPT–%BL 30 min	<0.001
Treatment	PPT–%BL 5 min	0.054
	PPT–%BL 30 min	0.214
Day	PPT–%BL 5 min	**0.**006**
	PPT–%BL 30 min	0.058
Treat*Day	PPT–%BL 5 min	0.902
	PPT–%BL 30 min	0.934
Corrected Model	PPi–%BL 5 min	0.775
	PPi–%BL 30 min	0.208
Intercept	PPi–%BL 5 min	<0.001
	PPi–%BL 30 min	<0.001
Treatment	PPi–%BL 5 min	0.207
	PPi–%BL 30 min	**0.**007**
Day	PPi–%BL 5 min	0.538
	PPi–%BL 30 min	0.627
Treat*Day	PPi–%BL 5 min	0.968
Corrected Model	PPu–%BL 5 min	0.089
	PPu–%BL 30 min	0.010
Intercept	PPu–%BL 5 min	<0.001
	PPu–%BL 30 min	<0.001
Treatment	PPu–%BL 5 min	**0.**005**
	PPu–%BL 30 min	***<**0**.**001**
Day	PPu–%BL 5 min	0.422
	PPu–%BL 30 min	0.566
Treat*Day	PPu–%BL 5 min	0.778
	PPu–%BL 30 min	0.949

**p < 0.01. ***p < 0.001. Abbreviations: PPT, pressure pain threshold; PPi, pressure pain intensity; PPu, pressure pain unpleasantness; %BL, percent baseline.

For the calf, results of follow-up univariate ANOVAs on QST are shown in Table 4. In contrast to our analysis of overall QST effects where we failed to find significant changes in pain measures for the calf baseline versus post-intervention, here we found a number of statistically significant effects of exercise on acute pain testing. We found that 30 min post-exercise constant heat intensity was significantly reduced by treatment (p = 0.004; Fig. 3D). We also found that 5 min post-exercise pressure pain threshold was significantly increased by treatment (p = 0.006; Fig. 3E) and 30 min post-exercise pressure pain threshold was significantly increased by day (p = 0.007; Fig. 3E).

**Table 4:** Acute effects of intervention on calf QST.

Source	Dependent Variable	ANOVA, P
Corrected Model	CHi–%BL 5 min	0.214
	CHi–%BL 30 min	0.137
Intercept	CHi–%BL 5 min	<0.001
	CHi–%BL 30 min	<0.001
Treatment	CHi–%BL 5 min	0.013
	CHi–%BL 30 min	**0.**004**
Day	CHi–%BL 5 min	0.443
	CHi–%BL 30 min	0.477
Treat*Day	CHi–%BL 5 min	0.941
	CHi–%BL 30 min	0.978
Corrected Model	PPT–%BL 5 min	0.034
	PPT–%BL 30 min	0.037
Intercept	PPT–%BL 5 min	<0.001
	PPT–%BL 30 min	<0.001
Treatment	PPT–%BL 5 min	**0.**006**
	PPT–%BL 30 min	0.192
Day	PPT–%BL 5 min	0.030
	PPT–%BL 30 min	**0.**007**
Treat*Day	PPT–%BL 5 min	0.966
	PPT–%BL 30 min	0.420

**p < 0.01. Abbreviations: CHi, constant heat intensity; PPT, pressure pain threshold; %BL, percent baseline.

## Discussion

This is the first study to (1) assess the dosing of a specific exercise training regimen on sensitivity to experimentally-induced pain and (2) to find evidence that repeated exercise can dose-dependently change perceived pain. The main findings of this study indicate that a moderate dose (5×/wk of 30 minutes/day) of moderate intensity walking (3.6 METs) is sufficient to reduce ratings of pressure pain in healthy subjects over a one-week period. Notably, although ratings of pressure pain decreased over 7 days, pressure threshold remained the same for all groups, and these effects were primarily seen in the forearm. To our knowledge, there are only six other primary studies that have specifically evaluated physical activity or exercise dose on pain in humans^12–17^. Since all of these other studies were limited to testing the effects of single acute bouts of exercise on acute pain perception or sensation, the critical question of exercise training dose in pain has been largely unanswered until now.

Pressure pain intensity and unpleasantness ratings for the forearm were significantly improved from the control group for the both the moderate (p < 0.01) and the high dose (p < 0.01) exercise intervention. However, no additional benefit was achieved from the high dose, suggesting a plateau effect. Such a plateau has been described for general health benefits beyond 45–60 minutes of daily moderate intensity exercise^19^. This suggests that for this exercise paradigm, the moderate dose is sufficient to achieve maximal analgesic effect, which is consistent with our hypothesis and exercise recommendations by the ACSM^9^. The choice of dose in this study was focused on frequency of training bouts per week, but other aspects of dose may be important in exercise dosing^20^. A recently published meta-analysis of exercise dosing in chronic pain by our group found that changing frequency is predicted to lead to the greatest changes in analgesic effect compared to changes in total minutes per week or study duration (number of weeks of study)^18^. Nonetheless, the potential variability in dosing of exercise therapy is likely to contribute significantly to differences between exercise studies.

Statistically significant differences were found for pressure pain VAS ratings of intensity and unpleasantness (i.e. pain perception), but not for pressure pain threshold for the forearm after one week of exercise training. These data suggest a disconnect between perception and sensation. Figure 1D represents a model for average pressure pain thresholds and subsequent average pain intensity ratings (VAS) for the respective threshold (connected by a black arrow) for the moderate and high dose exercise groups. For both groups, pressure pain threshold slightly increases from that measured at baseline; after the six-day intervention, the moderate and high dose groups rated their baseline pressure threshold substantially less painful than their previous rating. These data suggest that while this exercise paradigm was unable to significantly alter pain threshold, it significantly changed pain perception. Similar paradoxical effects have been shown in sustained aerobic exercise, where increases in pain tolerance were found despite no changes in pressure pain threshold following training^21^. Interestingly, this difference between threshold and perception is reversed for acute pain tests. Acute exercise data largely shows increases in pain threshold following acute bouts of exercise^22^. When we evaluated the acute effects of exercise in our study at the 5 min time point (post-exercise), we also found that pressure pain thresholds increased with exercise compared to control. Overall these data suggest alternative mechanisms are responsible for acute EIH versus sustained exercise training-induced changes in pain.

Somewhat surprisingly, we found robust analgesic effects shown in pre-post pressure ratings for the forearm, but no effect in the calf. One explanation for the null effect in the calf may be delayed onset muscle soreness (DOMS) having a masking effect on any analgesic effects of the exercise intervention. Irrespective of an individual’s general fitness level, physical activity of even a moderate intensity to which an individual is unaccustomed to can result in DOMS, which is known to persist for up to 5–7 days post-exercise^23^. We hypothesize that if this specific exercise program was continued past 7 days, the analgesic effects in the calf would be revealed. A second explanation for the null effect in the calf pressure data is due to the decreased number of sensory nociceptors and innervation density in the calf region as opposed to the forearm. Whole-body mapping of spatial acuity for pain has revealed that the volar surface of the forearm has dramatically lower two-point discrimination thresholds than the midcalf, indicating that the forearm is an area of higher spatial acuity for pain^24^. It is possible that the calf region was not sensitive enough to detect robust changes in pain perception. Robust effects in the forearm provide evidence of systemic hypoalgesia after exercise training, where pain perception decreased in the untrained limb. This agrees with previous findings of increases in pain tolerance in nonexercised limbs in healthy adults^21,25^.

Interestingly, the control group trended to have an increase in pressure pain intensity and unpleasantness ratings from baseline unlike the exercise groups, which exhibited overall decreases in pain ratings for the forearm (see Fig. 1A,B). It is possible that repeated probing of the pressure algometer to the same area of the forearm caused invisible micro-hematomas irrespective of treatment group. If this were the case, we might have expected hypersensitivity in pressure pain thresholds; this was not observed. We hypothesize that the repeat testing with the pressure algometer led to negative anticipation across patients leading to worsening perception of the stimulus. At the same time, the exercise regimen was able to reverse the increased pain perceptions in the exercise groups leading to an overall hypoalgesic benefit of exercise.

Although, no significant effects of treatment were found for percent change of HR, there are interesting trends indicating a training effect of exercise across the 3 sessions that all groups completed (Fig. 2). For the most part, measures of HR appear to decrease on each subsequent day of exercise training. Additionally, time-of-day has been shown to be a critical factor in regards to the exercise performance^26^. In the current study, a limited number of participants performed exercise around the same time each day. Due to the variability in the daily timing that subjects performed their exercise sessions, we were unable to parse out a time-of-day effect through analysis. However, previously research has shown significantly enhanced performance in subjects that performed exercise sessions in the evening compared to the morning, across various modalities^27–30^. This is important to consider in planning future studies.

A limitation to the present study included the initial lack of adequate randomization techniques. As noted in the methods, participants were randomly allocated to control, low and moderate dose exercise groups until the high dose group was later added to the study. This introduced a small source of selection bias in the study design. Due to the nature of the intervention, it was not possible to blind participants to their treatment group, which may contribute to performance bias in our study. Related to that, outcome assessors were not blinded to treatment group which may be a source of detection bias. Finally, each assay was performed in order of least invasive to most invasive to body tissue. Because the order of testing lacked randomization, this may lead to additional sources of biases on our outcomes.

This trial was represented by a fairly homogenous sample of participants demographically. This homogeneity also likely enhanced internal validity, reduced statistical and biologic variance and thus may have aided in the detection of significant differences. Although age inclusion was 18–40, the average age of participants was 21.6 years of age. Therefore, the implications of this study’s results may not be generalizable to older populations with chronic pain. Healthy female participants were included in the present analysis. Because of this, there may be unexplored sex differences in sensitivity to pain and pain perception related to exercise dose and the implications in different chronic pain conditions are unknown.

In conclusion, the results of this study demonstrated that a moderate dose (5×/wk of 30 min/day) of moderate intensity treadmill walking is sufficient to reduce perception of pressure pain in healthy individuals and that a higher dose does not necessarily provide any observable benefit in these measures. These results could provide a framework for developing a starting exercise training dose for individuals experiencing chronic musculoskeletal pain. This is one of the only studies to test different doses of exercise training on sensitivity to pain in healthy subjects or chronic pain participants. Additional studies must be done to determine appropriate prescription of exercise respective to exercise modality. This intervention is currently being tested in trials examining specific chronic pain populations.

## Methods

This study is registered with ClinicalTrials.gov under ID: NCT03642938 (08/22/2018).

### Participants

Forty healthy female subjects between the ages of 18 and 40 were recruited from the Duquesne University (Pittsburgh, PA, USA) community for this study. Study recruitment initially was open to both sexes, but later became exclusively female, due to unintended disproportionate recruiting of female participants. Nonetheless, we view this as a strength of the study in reducing biologic variability and still broadly informative given the higher prevalence of musculoskeletal pain conditions in females^31^. Inclusion criteria included (1) a normal BMI (18.5–25), (2) a resting heart rate between 60 and 100 bpm, and (3) a resting blood pressure less than or equal to 140/90 mmHg. Exclusion criteria included (1) a history of cardiac, respiratory, neurological or musculoskeletal disease, (2) acute pain, (3) chronic pain condition, (4) diabetes, (5) regular participation in high intensity athletic/sporting activities, (6) sedentary lifestyle, (7) anxiety or depression disorders, and (8) currently pregnant individuals.

### Study design

This study was a pseudo-randomized controlled trial on the effect of multiple doses of moderate intensity walking exercise on experimentally-induced pain following a 5-day intervention in healthy adults. This trial was pseudo-randomized between the control, low and moderate dose groups of exercise using a random number generator until data was collected for 19 total subjects, at which point the study was fully randomized in order to obtain sufficient power per group. Study coordinators (A.M.P & A.M.R.) were responsible for generating random allocation sequence, enrolling participants and assigning participants to interventions. A power analysis of pain outcome for control versus exercise indicated that a minimum of 10 people/group to be sufficient to detect statistical differences in our primary dependent variable (% baseline pain sensitivity) with an alpha of 0.05, power of 0.80, and effect size of 1.25 using data from the exercise literature. All procedures were approved by the Duquesne University Institutional Review Board (Protocol #2014-04-22) and written consent was obtained from each participant prior to testing. All methods were performed in accordance with the relevant international and local guidelines and regulations for human research.

### Procedures

Each trial was conducted at Duquesne University’s Exercise Physiology Laboratory over the course of 7 days. After screening and informed consent was obtained, participants were enrolled in the study. On the first day of testing, participants completed a series of questionnaires and underwent a sequence of quantitative and qualitative baseline sensitivity and pain assessments. Following baseline testing, treatment assignments were disclosed to the participants. One day following baseline assessments, subjects returned to the clinic to undergo their respective treatment assignment for the next 5 days. These sessions included either exercise or rest followed by pain assessment on days 1, 3 and 5 only. 24 hours following each subject’s final exercise session, pain and sensitivity thresholds were assessed. Subjects in the low dose exercise group performed exercise 3x per week; moderate group participants exercised 5x per week and high dose subjects performed exercise 10x per week (twice a day for 5 days). Subjects in the control condition rested during this time.

### Questionnaires

All participants completed four validated questionnaires pertaining to their general health, physical activity levels and anxiety levels. These included the AHA/ACSM Pre-participation Screening Form^32^, the Physical Activity Readiness Questionnaire (PAR-Q and YOU)^33^, the International Physical Activity Questionnaire (IPAQ-long)^34^, and the Social Interaction Anxiety Scale (SIAS)^35^.

### Exercise protocol

Subjects in exercise groups performed 30 minutes of moderate intensity walking exercise on a treadmill. Moderate intensity walking exercise was defined at the speed of 3.5 mph, at a 0% grade which falls between 3.0–5.9 MET range for moderate intensity physical activity. Prior to and following exercise, each experimental intervention participant rated their perceived exertion levels using the Borg RPE scale^36^. Each session began with a 2-minute warm-up at 2.5 mph and concluded with a 2-minute cool-down at 2.5 mph. In addition to monitoring perceived exertion, participants wore a heart rate monitor during exercise sessions in order to ensure exercise was being performed at a moderate-intensity. If heart rate was above 65% of predicted maximum heart rate, the treadmill speed was decreased. Participants in the low and moderate dose groups exercised for single 30-minute sessions each day (either 3 days total for the low group or 5 days total for the moderate group). Participants in the high dose group exercised for two 30-minute sessions per day with 5–10 minutes in between the two exercise sessions. It is important to note that the time of day that subjects performed exercise was not able to be controlled across groups or within subjects due to scheduling conflicts.

### Quantitative sensory testing (QST)

Quantitative sensory testing was done on the bare skin of the participant’s forearms and calves at specific testing sites, as previously shown^37^. For each test, the subjects were instructed to close their eyes and/or turn their head for the duration of the stimulus presentation. These assays assessed each subject’s cutaneous mechanical sensitivity (threshold for mechanical detection to 0.008 g, 0.02 g, 0.04 g, 0.07 g, 0.16 g, 0.4 g, 0.6 g and 1.0 g Touch Test filaments in 3 of 5 trials for filament), constant heat pain (45 °C 3 cm × 5 cm heating block (Benchmark Scientific) applied for 3 seconds followed by 10 cm Visual Analog Scale (VAS) for rating the intensity and unpleasantness of pain), radiant heat sensitivity (temperature ramp from 30 °C to 50 °C over 60 seconds with participant defined cutoff in temperature (°C) at “sensitivity threshold”; Thermal Stimulus Apparatus (IITC Woodland Hills, CA, USA) with custom heated glass), radiant heat pain (temperature ramp from 30 °C to 50 °C over 20 seconds with participant defined cutoff in temperature (°C) at “pain threshold”; Thermal Stimulus Apparatus), pressure pain threshold (1 cm round probe applied at constant ramping pressure until participant verbally defined cutoff in kg at “pain threshold”; Wagner Instruments, Greenwich, CT, USA) and constant pressure pain sensitivity (2 second pressure stimulus at participant defined threshold followed by VAS for rating the intensity of pain and unpleasantness of pain) as previously described^37^. 10 cm VAS scales were numbered at 0 and 10. Score was measured to the nearest mm. Testing was performed on baseline and 24 hours following the last exercise session to measure the overall change in sensitivity across the entire study. Testing was also performed on days 1, 3, and 5 of the trial at both 5 minutes and 30 minutes following exercise or rest to assess the acute effects of exercise on sensitivity and pain. One trial of each assay was performed per body site at each time point indicated. Testing was performed in the order listed above (least invasive to most invasive test) to avoid acute tissue damage and increased sensitization. Assays were performed on the subject’s forearms first, followed by testing on the calves. Each test was performed approximately 2 minutes apart to avoid desensitization of testing area.

### Statistical analysis

Prior to analysis, an *a priori* statistical plan was developed. Descriptive statistics were calculated using the IBM SPSS Version 25. Normality of the data was assessed. Nonparametric inferential statistics were used for data that were not normally distributed.

The primary outcome was defined as a change in QST 24 hours post-intervention versus baseline. This included a series of 8 quantitative sensory tests measured at baseline and at the completion of the 1-week intervention period. These tests were measured at two sites (forearm and calf). One-way ANOVAs were used to identify significant differences between groups, comparing percent change from baseline for parametric data. Given the number of statistical tests that were performed for the primary outcome measurements, a p-value of p < 0.01 was utilized for each body site (i.e. forearm or calf). Kruskal-Wallis Rank-sum tests were used for mechanical sensitivity at each site using p < 0.05. For analysis of primary outcomes, we were interested in looking at percent change, however the raw data values for constant pressure pain intensity and unpleasantness ratings for the forearm can be found as Supplementary Table S1 for full interpretation.

The secondary outcomes for this study include (1) HR during exercise, (2) Borg RPE before and after exercise and (3) measuring “acute” effects of exercise on QST testing. Two-way ANOVAs were used to examine percent change of HR and Borg RPE for the 3 treatment groups (low, moderate, high) from start of exercise (post-exercise/pre-exercise) across time (days 1, 3, 5) with an adjusted p-value of p < 0.03. Two-way MANOVAs were used to assess percent change from baseline (post-exercise/baseline test across the three days (day 1, 3, 5) and at the 5 minute and 30 minute time point between groups for the 8 QST). Analyses were grouped separately for tested body site. Given the number of statistical tests (n = 8) required for the QST secondary outcome measurements, a p-value of p < 0.01 was utilized for each body site.

The following demographic variables were collected and compared between groups to further check against potential bias: age, handedness, body mass index (BMI), baseline heart rate (HR), baseline blood pressure (BP), baseline IPAQ-long, and SIAS (social interaction anxiety scale). A significant difference in the proportion handedness was tested using the Chi-Square test where a p < 0.05 was considered significant. All other continuous variables were tested using a one-way ANOVA to test for significant differences between the four study groups (p < 0.05).

## Supplementary information

Consort Checklist

Dataset 1

## Data Availability

Raw data for this paper can be found at https://dsc.duq.edu/biology-data/1/.

## References

[1] Naugle KM, Fillingim RB, Riley JL (2012). A meta-analytic review of the hypoalgesic effects of exercise. *The journal of pain: official journal of the American Pain*. Society.

[2] Geneen LJ (2017). Physical activity and exercise for chronic pain in adults: an overview of Cochrane Reviews. The Cochrane database of systematic reviews.

[3] Steglitz J, Buscemi J, Ferguson MJ (2012). The future of pain research, education, and treatment: a summary of the IOM report “Relieving pain in America: a blueprint for transforming prevention, care, education, and research”. Translational behavioral medicine.

[4] Serpell M (2005). Pharmacological treatment of chronic pain. Anaesthesia & Intensive Care Medicine.

[5] Sullivan MD, Howe CQ (2013). Opioid therapy for chronic pain in the United States: promises and perils. Pain.

[6] Bhui K, Fletcher A (2000). Common mood and anxiety states: gender differences in the protective effect of physical activity. Social psychiatry and psychiatric epidemiology.

[7] Dunn AL, Trivedi MH, Kampert JB, Clark CG, Chambliss HO (2005). Exercise treatment for depression: efficacy and dose response. American journal of preventive medicine.

[8] Ambrose KR, Golightly YM (2015). Physical exercise as non-pharmacological treatment of chronic pain: Why and when. *Best practice & research*. Clinical rheumatology.

[9] Garber CE (2011). American College of Sports Medicine position stand. Quantity and quality of exercise for developing and maintaining cardiorespiratory, musculoskeletal, and neuromotor fitness in apparently healthy adults: guidance for prescribing exercise. Medicine and science in sports and exercise.

[10] Umeda M, Corbin LW, Maluf KS (2015). Examination of contraction-induced muscle pain as a behavioral correlate of physical activity in women with and without fibromyalgia. Disability and rehabilitation.

[11] Larsson C, Ekvall Hansson E, Sundquist K, Jakobsson U (2016). Impact of pain characteristics and fear-avoidance beliefs on physical activity levels among older adults with chronic pain: a population-based, longitudinal study. BMC geriatrics.

[12] Hoeger Bement MK, Dicapo J, Rasiarmos R, Hunter SK (2008). Dose response of isometric contractions on pain perception in healthy adults. Med Sci Sports Exerc.

[13] Ring C, Edwards L, Kavussanu M (2008). Effects of isometric exercise on pain are mediated by blood pressure. Biological psychology.

[14] Hoffman MD (2004). Intensity and duration threshold for aerobic exercise-induced analgesia to pressure pain. Archives of physical medicine and rehabilitation.

[15] Misra G, Paris TA, Archer DB, Coombes SA (2014). Dose-response effect of isometric force production on the perception of pain. PloS one.

[16] Koltyn KF, Arbogast RW (1998). Perception of pain after resistance exercise. British journal of sports medicine.

[17] Koltyn KF, Garvin AW, Gardiner RL, Nelson TF (1996). Perception of pain following aerobic exercise. Medicine and science in sports and exercise.

[18] Polaski AM, Phelps AL, Kostek MC, Szucs KA, Kolber BJ (2019). Exercise-induced hypoalgesia: A meta-analysis of exercise dosing for the treatment of chronic pain. PloS one.

[19] Simon HB (2015). Exercise and Health: Dose and Response, Considering Both Ends of the Curve. The American journal of medicine.

[20] Wasfy MM, Baggish AL (2016). Exercise Dose in Clinical Practice. Circulation.

[21] Jones MD, Booth J, Taylor JL, Barry BK (2014). Aerobic training increases pain tolerance in healthy individuals. Medicine and science in sports and exercise.

[22] Koltyn KF (2002). Exercise-induced hypoalgesia and intensity of exercise. Sports medicine.

[23] Armstrong RB (1984). Mechanisms of exercise-induced delayed onset muscular soreness: a brief review. Medicine and science in sports and exercise.

[24] Mancini F (2014). Whole-body mapping of spatial acuity for pain and touch. Annals of neurology.

[25] Anshel MH, Russell KG (1994). Effect of aerobic and strength training on pain tolerance, pain appraisal and mood of unfit males as a function of pain location. Journal of sports sciences.

[26] Duglan D, Lamia KA (2019). Clocking In, Working Out: Circadian Regulation of Exercise Physiology. Trends in endocrinology and metabolism: TEM.

[27] Bessot N (2006). The effect of pedal rate and time of day on the time to exhaustion from high-intensity exercise. Chronobiology international.

[28] Chtourou H, Souissi N (2012). The effect of training at a specific time of day: a review. Journal of strength and conditioning research / National Strength & Conditioning Association.

[29] Fernandes AL (2014). Effect of time of day on performance, hormonal and metabolic response during a 1000-M cycling time trial. PloS one.

[30] Dalton B, McNaughton L, Davoren B (1997). Circadian rhythms have no effect on cycling performance. International journal of sports medicine.

[31] Fillingim RB, King CD, Ribeiro-Dasilva MC, Rahim-Williams B, Riley JL (2009). Sex, gender, and pain: a review of recent clinical and experimental findings. The journal of pain: official journal of the American Pain Society.

[32] Balady GJ (1998). Recommendations for cardiovascular screening, staffing, and emergency policies at health/fitness facilities. Circulation.

[33] Thomas S, Reading J, Shephard RJ (1992). Revision of the Physical Activity Readiness Questionnaire (PAR-Q). Can J Sport Sci.

[34] Craig CL (2003). International physical activity questionnaire: 12-country reliability and validity. Med Sci Sports Exerc.

[35] Mattick RP, Clarke JC (1998). Development and validation of measures of social phobia scrutiny fear and social interaction anxiety. Behaviour research and therapy.

[36] Borg GA (1982). Psychophysical bases of perceived exertion. Medicine and science in sports and exercise.

[37] Kostek, M. *et al*. A protocol of manual tests to measure sensation and pain in humans. *Journal of visualized experiments: JoVE*, 10.3791/54130 (2016). 10.3791/54130 PMC5226435 28060280

