# Supplementary material for: The dosing of aerobic exercise therapy on experimentally-induced pain in healthy female participants
Source: Sci Rep. 2019 Oct 16;9:14842. doi: 10.1038/s41598-019-51247-0 (PMC6795887; doi:10.1038/s41598-019-51247-0)
Supplement: Supplementary file 2 — Dataset 1 [file 41598_2019_51247_MOESM2_ESM.docx]

**The dosing of aerobic exercise therapy on experimentally-induced pain in healthy female participants**.

Anna M. Polaski^1,2^, Amy L. Phelps^2,3^, Kimberly A. Szucs^2,4^, Austin M. Ramsey^1,2^, Matthew C. Kostek^2,5^, and Benedict J. Kolber^1,2,*^

^1^Department of Biological Sciences, Duquesne University, Pittsburgh, Pennsylvania, United States; ^2^Chronic Pain Research Consortium, Duquesne University, Pittsburgh, Pennsylvania, United States; ^3^Palumbo Donahue School of Business, Statistics, Duquesne University, Pittsburgh, Pennsylvania, United States; ^4^Department of Occupational Therapy, Duquesne University, Pittsburgh, Pennsylvania, United States; ^5^Department of Physical Therapy, Duquesne University, Pittsburgh, Pennsylvania, United States.

**Supplemental Material File Listing**:
Supplemental Table 1

**Corresponding Author:**

Benedict Kolber, Duquesne University, 600 Forbes Ave, Pittsburgh, Pennsylvania, United States; kolberb@duq.edu; telephone: 412-396-5615; fax: 412-396-5907

|  | **Baseline**  *Constant Pressure VAS* | | **Post-intervention**  *Constant Pressure VAS* | |
| --- | --- | --- | --- | --- |
|  | Intensity | Unpleasantness | Intensity | Unpleasantness |
| **Control** | 0.7 | 1.5 | 1.8 | 1.8 |
|  | 1.3 | 3.4 | 2.9 | 4.8 |
|  | 3.1 | 1.4 | 2.2 | 3.2 |
|  | 1.2 | 2.1 | 2.6 | 4.2 |
|  | 5.9 | 5.4 | 4.8 | 3.1 |
|  | 1.6 | 3.7 | 3.0 | 4.3 |
|  | 2.2 | 1.1 | 2.5 | 2.0 |
|  | 0.9 | 3.2 | 1.1 | 1.9 |
|  | 2.6 | 4.9 | 5.1 | 5.7 |
|  | 3.9 | 5.9 | 4.2 | 3.4 |
| **Low dose** | 1.6 | 3.4 | 2.2 | 4.0 |
|  | 2.6 | 3.3 | 4.0 | 4.2 |
|  | 2.9 | 1.9 | 5.4 | 4.2 |
|  | 3.3 | 5.0 | 2.9 | 3.6 |
|  | 6.9 | 5.9 | 4.5 | 3.5 |
|  | 0.8 | 0.7 | 0.4 | 0.3 |
|  | 2.7 | 3.6 | 3.4 | 4.0 |
|  | 3.5 | 3.0 | 1.2 | 2.3 |
|  | 7.6 | 7.1 | 2.2 | 2.4 |
|  | 6.2 | 7.2 | 3.7 | 4.4 |
| **Moderate dose** | 0.9 | 0.5 | 0.2 | 0.1 |
|  | 2.8 | 2.5 | 1.6 | 1.1 |
|  | 4.3 | 5.3 | 0.4 | 0.5 |
|  | 0.8 | 2.1 | 0.6 | 0.6 |
|  | 3.5 | 4.4 | 1.5 | 1.8 |
|  | 2.4 | 4.4 | 1.1 | 2.7 |
|  | 2.3 | 2.5 | 1.1 | 1.1 |
|  | 2.7 | 4.5 | 2.0 | 4.0 |
|  | 2.0 | 1.0 | 1.0 | 0.3 |
|  | 6.8 | 7.5 | 4.9 | 7.6 |
| **High dose** | 2.0 | 3.4 | 0.3 | 0.5 |
|  | 3.9 | 4.2 | 2.0 | 1.6 |
|  | 3.2 | 4.0 | 0.9 | 0.9 |
|  | 0.4 | 1.5 | 0.9 | 0.9 |
|  | 5.6 | 4.3 | 0.7 | 0.5 |
|  | 1.8 | 1.6 | 0.1 | 0.1 |
|  | 1.9 | 1.4 | 0.1 | 0.7 |
|  | 1.5 | 1.8 | 3.1 | 3.0 |
|  | 3.8 | 6.7 | 2.0 | 2.9 |
|  | 1.7 | 1.9 | 1.2 | 1.0 |

**Supplemental Table 1**. Raw data values of participants’ baseline and post-intervention measures for the constant pressure pain ratings assay as tested on the forearm.
